# Supplementary material for: Cucumber Mildew Resistance Locus O Interacts with Calmodulin and Regulates Plant Cell Death Associated with Plant Immunity
Source: Int J Mol Sci. 2019 Jun 19;20(12):2995. doi: 10.3390/ijms20122995 (PMC6627319; doi:10.3390/ijms20122995)
Supplement: Supplementary file 1 [file ijms-20-02995-s001.pdf]

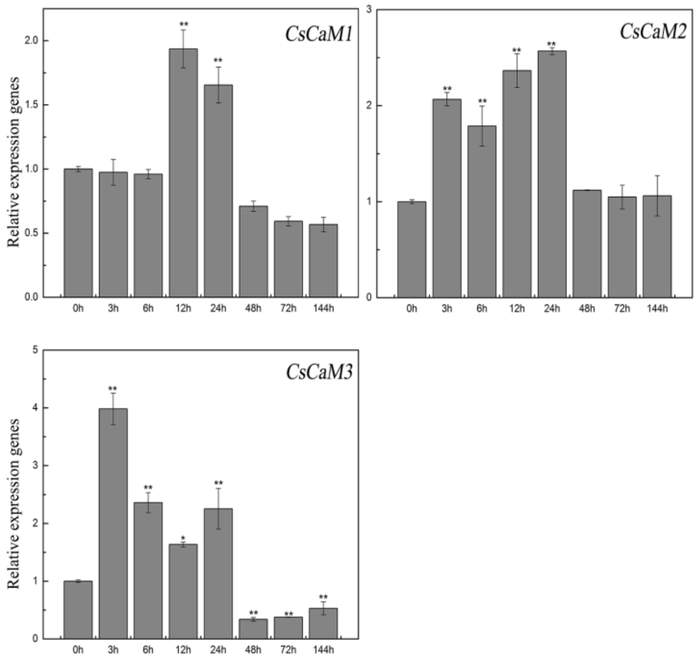

Figure S1. Expression patterns of C*sCaMs* in Xintaimici cultivars were inoculated with *C. cassiicola.* Expression analysis of candidate genes at 0, 3,6, 12, 24, 48, 72, and 144 hpi (hours post-inoculation) using the $2^{-\Delta\Delta Ct}$ method. Data are the means ± standard deviations from three biological experiments. The asterisks indicate a significant difference (Student's *t* test, **P* < 0.05 or ***P* < 0.01).

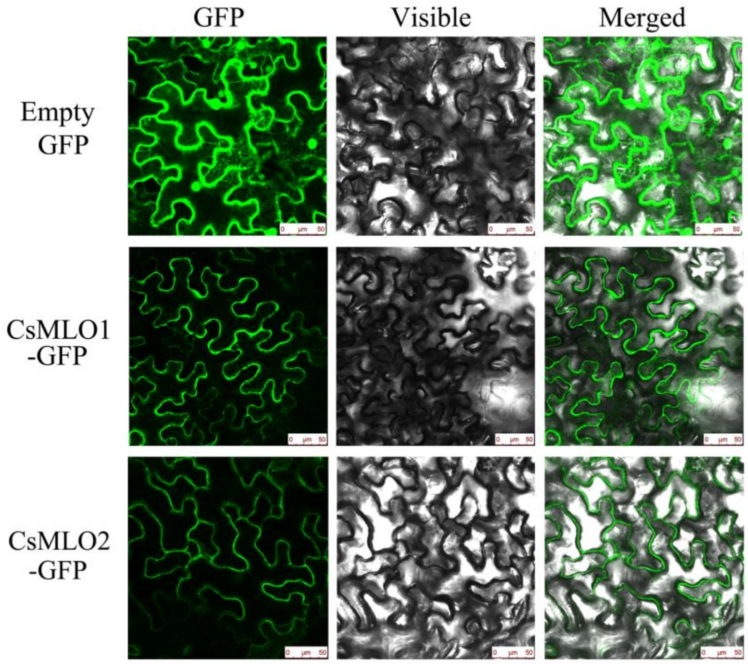

Figure S2. Subcellular localization of CsMLO1 and CsMLO2 in *N. benthamiana* leaf cells. The green fluorescent protein (GFP) alone localized throughout the whole cell while CsMLO1-GFP and CsMLO2-GFP localized in the plasma membrane. Green fluorescence images (right) were obtained at 48 h via Leica confocal microscopy after agroinfiltration. Bars = 50 μm.

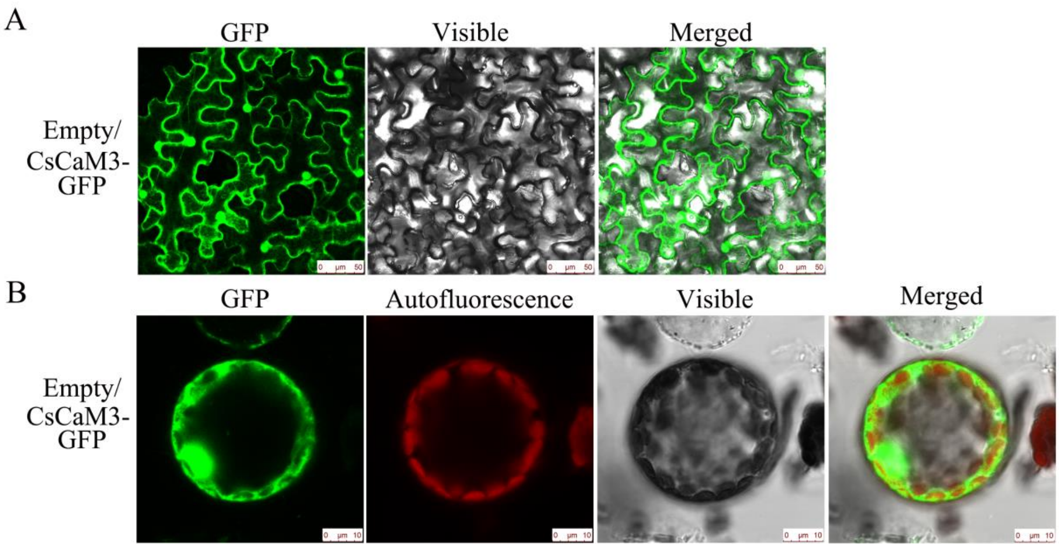

Figure S3. Subcellular localization analyses of empty (35S::GFP)+CsCaM3-GFP in transiently transformed *N. benthamiana* epidermal cells and protoplasts. Confocal images of green fluorescent of empty+CsCaM3-GFP were obtained in *N. benthamiana* epidermal cells 3 days after *Agroinfiltration*. Chloroplast autofluorescence (red). Bars = 50 μm.

Table S1. List of primers used in the study.

Analysis	Primer name	Sequence(5′-3′)
qRT-PCR for *CsMLO1*	*RTCsMLO1*-F	ATGCGTTGGCTAGAGCTAAGATG
	RTCsMLO1-R	AGGTGTCTCTTGCAAACCTAAACC
qRT-PCR for *CsMLO2*	*RTCsMLO2*-F	GTCATTCACCTCACTGGAAAGTGG
	RTCsMLO2-R	AGTGACAGCATCTTGGCCTATCG
CsCaM1 for qRT-PCR	*qCsCaM1*-F	CTGTGTTTGTGACCCTTTGAAT
	qCsCaM1-R	GAGGAACAGCAAAGACACTTTT
CsCaM2 for qRT-PCR	*qCsCaM2*-F	TAGATGAGATGATTCGTGAGGC
	qCsCaM2-R	AAAGAAGGGGTTTCTCCTACAG
CsCaM3 for qRT-PCR	*qCsCaM3*-F	GGCAAGAAAAATGAAGGACACT
	qCsCaM3-R	GATATCGAAATCAACGCCATCC
Cucumber *CsActin* gene for and qRT-PCR	Actin-F	TCGTGCTGGATTCTGGTG
	Actin-R	GGCAGTGGTGGTGAACAT
CsMLO1-silencing vector	*TRVCsMLO1*-F	GAATTCGTGGCAGAGGCCCTTCGCAAC
	TRVCsMLO1-R	GAGCTCTCATTCAACTCTATCAAATGA
CsMLO2-silencing vector	*TRVCsMLO2*-F	GTGAGTAAGGTTACCGAATTCCACCACTCAGCCAAGAAG
	TRVCsMLO2-R	GGCCTCGAGACGCGTGAGCTCTCATTTGGCAAATGAGAA
CsMLO1-overexpression vector	*CsMLO1*-F	TTGATACATATGCCCGTCGACATGGCGGGGGCAGCCGGTGG

	CsMLO1-R	GCCCTTGCTCACCATGGATCCTTCAACTCTATCAAATGAAA
CsMLO2-overexpression vector	*CsMLO2*-F	TTGATACATATGCCCGTCGACATGGCTGAATGTGGAACAGA
	CsMLO2-R	GCCCTTGCTCACCATGGATCCTTTGGCAAATGAGAAGTCTG
Chimeric primer for *CsMLO1*-GFP	*CsMLO1*-GFP-F	CCGATCGTTGGGACAACG
	CsMLO1-GFP-R	AGGGCACGGGCAGCTTGC
Chimeric primer for *CsMLO2*-GFP	*CsMLO2*-GFP-F	GTTGGGAAGAGTTGCCTCC
	CsMLO2-GFP-R	AGGGCACGGGCAGCTTGC
CsCaM1-overexpression vector	*CsCaM1*-F	TTGATACATATGCCCGTCGACATGGCGGATCAGCTAACCGATG
	CsCaM1-R	GCCCTTGCTCACCATGGATCCCTTGGCCATCATGACCTTCAC
CsCaM2-overexpression vector	*CsCaM2*-F	TTGATACATATGCCCGTCGACATGGCTGATCAGCTCACCGACG
	CsCaM2-R	GCCCTTGCTCACCATGGATCCCTTGGCCATCATGACTTTC
CsCaM3-overexpression vector	*CsCaM3*-F	TTGATACATATGCCCGTCGACATGGCCGAGCAGCTCACCGAC
	CsCaM3-R	GCCCTTGCTCACCATGGATCCCTTGGCCATCATGATTTTTACG

Table S2. PCR primers used in this study.

Analysis	Primer name	Sequence(5′-3′)
CsMLO1 (CaMBD) for Yeast two-Hybrid Assay	CsMLO1-BD-F	ATGGCCATGGAGGCCGAATTCATGGCAGAGGCCCTTCGCAAT
	CsMLO1-BD-R	CCGCTGCAGGTCGACGGATCCTCAGCGGTTGTGTTTGATGTG
CsMLO2 (CaMBD) for Yeast two-Hybrid Assay	CsMLO1-BD-F	ATGGCCATGGAGGCCGAATTCATGGCAACGGCATTGAAGAAC
	CsMLO1-BD-R	CCGCTGCAGGTCGACGGATCCTCAGCGGTGCTGCTTCATGTT
CsCaM1 for Yeast two-Hybrid Assay	*CsCaM1*-F	GAGGCCAGTGAATTCATGGCGGATCAGCTAACCGATG
	CsCaM1-R	GAGCTCGATGGATCCCTTGGCCATCATGACCTTCAC
CsCaM2 for Yeast two-Hybrid Assay	*CsCaM2*-F	GAGGCCAGTGAATTCTCAATGGCTGATCAGCTCACCGACG
	CsCaM2-R	GAGCTCGATGGATCCTCACTTGGCCATCATGACTTTC

CsCaM3 for Yeast two-Hybrid Assay	*CsCaM3*-F	GAGGCCAGTGAATTCATGGCCGAGCAGCTCACCGAC
	CsCaM3-R	GAGCTCGATGGATCCTCACTTGGCCATCATGATTTTTA
CsCaM1 for LUC Imaging Assay	cLUC-CaM1-HA-F	TACGCGTCCCGGGGCGGTACCATGGCGGATCAGCTAACCG
	cLUC-CaM1-HA-R	TCACGCATAGTCAGGAACATCGTAAGGGTACTTGGCCATCATGACCTTCAC
CsCaM2 for LUC Imaging Assay	cLUC-CaM2-HA-F	TACGCGTCCCGGGGCGGTACCATGGCTGATCAGCTCACCGA
	cLUC-CaM2-HA-R	TCACGCATAGTCAGGAACATCGTAAGGGTACTTGGCCATCATGACTTTCAC
CsCaM3 for LUC Imaging Assay	cLUC-CaM3-HA-F	TACGCGTCCCGGGGCGGTACCATGGCCGAGCAGCTCACC
	cLUC-CaM3-HA-R	TCACGCATAGTCAGGAACATCGTAAGGGTACTTGGCCATCATGATTTTTACG
LUC Imaging Assay	HA-RR	ACGAAAGCTCTGCAGGTCGACTCACGC
CsMLO1 (CaMBD) for LUC Imaging Assay	Flag-MLO1-cLUC-F	ACGGGGGACGAGCTCGGTACCATGG
	Flag-MLO1-cLUC-R	CGCGTACGAGATCTGGTCGACGCGGTT
CsMLO1 (CaMBD) for LUC Imaging Assay	Flag-MLO2-cLUC-F	ACGGGGGACGAGCTCGGTACCATGG
	Flag-MLO2-cLUC-R	CGCGTACGAGATCTGGTCGACGCGGTG

Table S3. PCR primers used in this study.

Analysis	Primer name	Sequence (5′-3′)
CsMLO1 for BIFC analysis	BiFC-MLO1-NF	GGGGACAAGTTTGTACAAAAAAGCAGGCTTCGGTACCATGGCAGAGGCCCTTCGC
	BiFC-MLO1-NR	GGGGACCACTTTGTACAAGAAAGCTGGGTGACTAGTGCGGTTGTGTTTGATGTG
CsMLO2 for BIFC analysis	BiFC-MLO2-NF	GGGGACAAGTTTGTACAAAAAAGCAGGCTTCGGTACCATGGCGACGGCATTGAAG
	BiFC-MLO2-NR	GGGGACCACTTTGTACAAGAAAGCTGGGTGACTAGTGCGGTGCTGCTTCATGTTC
CsCaM1 for BIFC analysis	BiFC-CaM1-CF	GGGGACAAGTTTGTACAAAAAAGCAGGCTTCGGTACCATGGCGGATCAGCTAACCG

	BiFC-CaM1-CR	GGGGACCACTTTGTACAAGAAAGCTGGGTG ACTAGTCTTGGCCATCATGACCTTCAC
CsCaM2 for BIFC analysis	BiFC-CaM2-CF	GGGGACAAGTTTGTACAAAAAAGCAGGCTT CGGTACCATGGCTGATCAGCTCACCGA
	BiFC-CaM2-CR	GGGGACCACTTTGTACAAGAAAGCTGGGTG ACTAGTCTTGGCCATCATGACTTTCAC
CsCaM3 for BIFC analysis	BiFC-CaM3-CF	GGGGACAAGTTTGTACAAAAAAGCAGGCTT CGGTACCATGGCCGAGCAGCTCACC
	BiFC-CaM3-CR	GGGGACCACTTTGTACAAGAAAGCTGGGTG ACTAGTCTTGGCCATCATGATTTTTACG
CsMLO1 for Subcellular co-localization	pRI101-MLO1-F	CATATGCCCGTCGACATGGCGGGGGCAGCC GGT
	pRI101-MLO1-R	TCAGAATTCGGATCCTCATTCAACTCTATCA AATG
CsMLO2 for Subcellular co-localization	pRI101-MLO2-F	CATATGCCCGTCGACATGGCTGAATGTGGA ACAGAACATCAGAATTCGGATCCTCATTTG GCAAATGAGAAGTC
	pRI101-MLO2-R	
